# Are malaria rapid diagnostic test results stable over time to support verification of surveillance data?

**DOI:** 10.1186/s12936-025-05595-0

**Published:** 2025-10-22

**Authors:** Corine Ngufor, Kim A. Lindblade, Sunday Atobatele, Arthur Mpimbaza, Idelphonse Ahogni, Nelson Ssewante, Ese Akpiroroh, Augustin Kpemasse, Onyebuchi Okoro, Bosco Agaba, Shawna Cooper, Kevin Griffith, Michael Humes

**Affiliations:** 1 Centre de Recherche Entomologique de Cotonou, Cotonou, Benin; 2 PMI Insights Project/PATH, Geneva, Switzerland; 3 Sydani Group, Abuja, Nigeria; 4 Child Health and Development Centre, Makerere University, Kampala, Uganda; 5 Programme National de Lutte Contre Le Paludisme, Cotonou, Benin; 6 National Malaria Elimination Programme, Abuja, Nigeria; 7 National Malaria Control Division, Kampala, Uganda; 8 Audere, Seattle, WA USA; 9 United States President’s Malaria Initiative, Washington, DC USA

**Keywords:** Malaria, Rapid diagnostic tests, Surveillance, Benin, Nigeria, Uganda, Stability

## Abstract

**Background:**

Rapid diagnostic tests (RDTs) have improved malaria case management by enabling point-of-care confirmation of infection, particularly in low-resource settings. In addition to clinical use, RDT results recorded in health facility registers are a critical component of national malaria surveillance systems. Recently, national programmes have explored using stored RDT cassettes to validate register data. However, manufacturers caution that results should be read within 15–30 min, raising concerns about result validity after this period. This study evaluated the stability of RDT results over a one-month period to assess whether stored cassettes can reliably reflect initial test outcomes.

**Methods:**

A prospective, observational study was conducted in 48 health facilities across Benin, Nigeria, and Uganda from June to September 2023. A digital artificial intelligence (AI)-powered RDT reader (HealthPulse, Audere, Seattle WA USA) was used to photograph RDTs immediately after interpretation by health workers and again at one week and one month. RDTs were stored under typical health facility conditions during the study. Images were independently interpreted by a trained panel, with results categorized as positive, negative, invalid, or uninterpretable. Only RDTs with valid interpretations at all three time points were included in the final analysis. Positive and negative predictive values (PPV and NPV) were calculated to measure the accuracy of results from stored RDTs relative to the initial interpretation.

**Results:**

Out of 54,251 RDTs captured, 45,155 (83.2%) met inclusion criteria. At one month, 95.1% of initially positive RDTs remained positive, and 95.3% of initially negative RDTs remained negative. The PPV of a positive result at one month was 96.3% (95% CI 96.1, 96.5), while the NPV of a negative result was 93.8% (95% CI 93.4, 94.1). Most result changes occurred within the first week. Faint lines were associated with higher rates of change in both directions; 26.8% changing from positive to negative and 48.1% changing from negative to positive. Stability of results also varied across RDT products and specific test lines.

**Conclusions:**

Stored RDT cassettes maintain high result stability over one month and can serve as a reliable reference to verify health facility records. Result changes were linked to premature interpretation, faint lines or product- or line-specific characteristics. Adherence to manufacturer-recommended read times may reduce the proportion of RDTs that change from negative to positive. These findings support the utility of stored RDTs in improving data quality and rational antimalarial use in malaria-affected settings.

**Supplementary Information:**

The online version contains supplementary material available at 10.1186/s12936-025-05595-0.

## Background

Rapid diagnostic tests (RDTs) have transformed malaria case management in resource-limited settings by enabling point-of-care confirmation of infection prior to treatment. They require minimal expertise, no refrigeration or specialized equipment and no electricity, making them suitable for use in a wide range of environments. RDT results recorded in health facility registers also underpin malaria surveillance systems in endemic countries, providing essential data for tracking disease trends, guiding resource allocation, and assessing the effectiveness of control measures [1].

Malaria RDTs are lateral flow immunoassays that use antibodies to detect specific antigens produced by malaria parasites in the bloodstream. These antibodies are immobilized on a nitrocellulose strip housed within a plastic cassette. A few drops of blood from a finger prick are placed into one well of the cassette and buffer solution is added to a second well. The buffer lyses red blood cells, releasing any parasite proteins. Dye-labelled antibodies specific for one or more *Plasmodium* species then bind to parasite antigens. Capillary action moves the blood and antigen–antibody complexes along the membrane, where they are captured by one or more lines of fixed antibodies (the T, or test, lines), forming visible colored bands in the results window. The control (C) line, located further down the membrane, captures excess dye-labelled antibodies and forms a visible colored band that indicates the test has functioned correctly.

Malaria RDTs primarily target two antigens: histidine-rich protein 2 (HRP2) and *Plasmodium* lactate dehydrogenase (pLDH), with aldolase used less commonly. HRP2 is specific to *Plasmodium falciparum*, while pLDH is produced by all human-infecting *Plasmodium* species. [2]. The most commonly used RDTs feature a single test line that detects HRP2, followed by formats with two test lines, where the second line detects either pan-pLDH or *Plasmodium vivax*-specific pLDH [3]. Both HRP2 and pLDH lines can appear faint at low parasite densities; however, for a given parasite density, the pLDH line is typically less intense than the HRP2 line. Interestingly, HRP2 lines may also appear weak at very high parasite densities [4, 5].

More than 328 million RDTs were performed globally in 2023 [6]. The vast majority took place in the World Health Organization (WHO) African Region (266 million, 81%) and the South-East Asia Region (44 million, 14%). In Africa, RDTs are used nearly four times more often than microscopy for malaria diagnosis. Despite this heavy reliance on RDTs for confirming infection, many healthcare workers (HCWs) do not consistently base treatment decisions on RDT results [7–9] mostly choosing to rely on clinical presentation rather than test outcomes. As a result, discrepancies between test outcomes and clinical decisions can lead to inaccurate recording of RDT results in health facility registers [10].

In 2023, the Ministry of Health of Benin launched monthly data validation meetings at the district level as part of a national strategy to enhance the accuracy, completeness, and timeliness of routine malaria data reporting [11]. A key innovation introduced through this process was the systematic verification of patient register entries by comparing them to the physical results still visible on used and archived RDT cassettes that were stored at health facilities. This verification method offered a practical means of cross-checking whether the test outcomes recorded in facility registers accurately reflected the actual diagnostic results. However, it also raised important technical questions regarding the stability and reliability of RDT results over time. RDT manufacturers typically recommend that results be interpreted within a short time window (typically 15–30 min after sample application), because the chemical reactions that produce the visible lines may degrade or change beyond this window, potentially leading to misinterpretation of results [12]. Given the potential usefulness of Benin’s retrospective malaria data validation approach using archived RDT cassettes, this study assessed whether RDT results could be reliably interpreted up to one month after testing. To guide future data validation efforts using archived RDT cassettes, a dedicated study was conducted as part of a broader multicountry evaluation on the accuracy of RDT records in health facilities. The objective was to assess the stability and interpretability of RDT results over a one-month period of storage under typical health facility conditions.

## Methods

This evaluation was conducted from June to September 2023 in the context of a larger study that has been described elsewhere [10]. Briefly, a prospective, observational study was performed in selected health facilities across Benin, Nigeria, and Uganda (Côte d’Ivoire was included in the main study but did not participate in this assessment due to resource constraints).

Trained research assistants were present during principal operating hours in 16 health facilities in each country and used a digital artificial intelligence (AI)-powered RDT reader (HealthPulse, Audere, Seattle, WA USA) to take photographs of all RDTs performed in the study facilities. The HealthPulse RDT reader has an image quality assurance component that leverages computer vision and machine learning processes to assess the quality of images of RDTs, immediately flagging those that do not meet quality standards (such as those with blur or multiple RDTs) and prompting the user to retake the photo. The HealthPulse application includes an AI algorithm running on the mobile device that interprets the RDT result from the image with demonstrated high accuracy of 96.8%.

Research assistants photographed RDTs as soon as possible after they were interpreted by HCWs; however, the timing of interpretation was determined by the HCWs, and the time elapsed between sample application and result interpretation was not recorded. Each RDT was labelled with a unique, preprinted barcode placed on the back. A paired label was placed against the patient data in the health facility register and research assistants recorded the HCW interpretation of the RDT.

During the first three months of the study, used RDT cassettes were placed in paper envelopes and stored in cardboard boxes at health facilities under ambient temperature and humidity conditions. The RDTs were re-photographed at one week and again at one month following the initial interpretation. At each time point, the barcode on the back of the cassette was scanned to allow images to be matched over time. The HealthPulse application recorded the date and time of each image capture. After the final image capture, RDT cassettes were discarded in accordance with national disposal guidelines.

All RDT images were stripped of metadata and sent to an external, quality-controlled panel trained in interpreting RDT results from images. The durability of the RDT results was determined from the trained panel’s interpretation of individual RDT images at each timepoint. For RDT products with two test lines, the HRP2 line is referred to as T0 while the pLDH line is referred to as T1.

The panel classified each line on the test as present, absent, or obstructed from view, and a simple algorithm categorized each RDT result as positive (C line present and one or more T lines present), negative (C line present and no T lines present and no T lines obstructed from view), invalid (C line absent and not obstructed from view), or uninterpretable (lines obstructed from view). The panel noted the RDT product name (i.e. brand and model, Supplemental Table 1), flagged images of RDTs with excess blood in the result window and noted the presence of faint test lines, although they did not note which test line was faint. All RDT result classifications and observations on presence of lines at each time point were determined by the external panel.

**Table 1:** Characteristics of rapid diagnostic tests stored and followed up to one month

Characteristic	Total N = 44,605 n (%)	Benin N = 15,334 n (%)	Nigeria N = 6718 n (%)	Uganda N = 22,553 n (%)
Initial result				
Positive	25,129 (56.3)	8868 (57.8)	2645 (39.4)	13,616 (60.4)
Negative	19,420 (43.5)	6461 (42.1)	4070 (60.6)	8889 (39.4)
Invalid	56 (0.1)	5 (0.0)	3 (0.0)	48 (0.2)
One-week result				
Positive	25,022 (56.1)	8691 (56.7)	2968 (44.2)	13,363 (59.3)
Negative	19,575 (43.9)	6640 (43.3)	3750 (55.8)	9185 (40.7)
Invalid	8 (0.0)	3 (0.0)	0 (0.0)	5 (0.0)
One-month result				
Positive	24,864 (55.7)	8697 (56.7)	2855 (42.5)	13,312 (59.0)
Negative	19,733 (44.2)	6633 (43.3)	3863 (57.5)	9237 (41.0)
Invalid	8 (0.0)	4 (0.0)	0 (0.0)	4 (0.0)
RDT product				
AdvDx Malaria Pf	6711 (15.0)	0 (0.0)	6704 (99.8)	7 (0.0)
Bioline Malaria Pf	27,846 (62.4)	15,127 (98.7)	14 (0.2)	12,705 (56.3)
Bioline Malaria Pf (HRP2/pLDH)	201 (0.5)	0 (0.0)	0 (0.0)	201 (0.9)
First Response Malaria Pf	1395 (3.1)	0 (0.0)	0 (0.0)	1395 (6.2)
First Response Malaria Pf Ag (pLDH/HRP2)	1951 (4.4)	0 (0.0)	0 (0.0)	1951 (8.7)
ParaHIT Pf	322 (0.7)	1 (0.0)	0 (0.0)	321 (1.4)
Standard Q Pf	626 (1.4)	206 (1.3)	0 (0.0)	420 (1.9)
Other	322 (0.7)	1 (0.0)	0 (0.0)	321 (1.4)
Faint line on initial result				
Yes	4246 (9.5)	651 (4.2)	644 (9.6)	2951 (13.1)
No	40,359 (90.5)	14,683 (95.8)	6074 (90.4)	19,602 (86.9)
Blood obscuring results window on initial result				
Yes	6 (0.0)	0 (0.0)	0 (0.0)	6 (0.0)
No	44,599 (100)	15,334 (100)	6718 (100)	22,547 (100)

*HRP2* histidine-rich protein 2, *Pf* *Plasmodium falciparum*, *pLDH* *Plasmodium* lactate dehydrogenase, *RDT* rapid diagnostic test

### Data management and analysis

As this was an exploratory, descriptive study, no sample size calculations were performed. Observations were excluded if the initial RDT result was uninterpretable, as these were considered to reflect problems with the image rather than true test outcomes. For analyses involving individual test lines, RDTs in which one or more test lines were judged to be obstructed from view were excluded.

Images taken between five and 10 days (inclusive) after the initial image were classified as ‘one week,’ while those taken between 25 and 35 days (inclusive) were classified as ‘one month.’ If multiple images were captured within the same time window, the first image was retained. The final analytical data set included only records with results for all time points.

Simple descriptive statistics were conducted in R (R Foundation for Statistical Computing, Vienna, Austria). The positive and negative predictive values (PPV and NPV, respectively) for results at one month were calculated to measure the probability that they accurately reflected the initial results, and 95% confidence intervals were calculated using the Wilson score method for binomial proportions using the *binom* package in R [13].

### Ethical issues

No patients were consented by the study team as the RDT images were recorded after patient consultation was concluded and without any accompanying personal identifying information. The PATH institutional review board approved the multi-country study protocol. In Benin, the Comité National d’Ethique pour la Recherche en Santé provided approval. In Nigeria, approval was received from Oyo State Ministry of Health Research Ethics Committee, Sokoto State Health Research Ethics Committee and the National Health Research Ethics Committee of Nigeria. The Uganda National Council for Science and Technology and Vector Control Division-Research & Ethics Committee both reviewed and approved the study in Uganda.

## Results

From June through September 2023, 154,141 RDT images representing 54,251 RDTs were collected. After excluding 9130 RDT images that were missing one or more follow-up images, the analytical database included 44,605 RDTs (82.2%).

### Exclusion due to missing follow-up images

Among the RDT images excluded due to missing follow-up images, the majority (5374, 58.9%) were initially photographed in September and were scheduled for follow-up after the evaluation had concluded. To assess whether there was any bias associated with missing follow-up images, the 31,907 RDTs photographed before August were analysed, of which 1336 (4.2%) were missing one or more follow-up images. RDTs initially interpreted as negative were more likely to miss follow-up images (748, 5.7%) than those initially interpreted as positive (581, 3.1%). The proportion of RDTs missing follow-up images was higher in Nigeria (638, 16.2%) compared to Uganda (534, 3.2%) and Benin (164, 1.5%). However, the presence of faint lines was not associated with missing follow-up images (with faint line: 131, 4.2%; without faint line: 1205, 4.2%).

### RDT characteristics

Uganda contributed 22,553 (50.6%), Benin 15,334 (33.4%) and Nigeria 6718 (15.1%) RDTs (Table 1). The panel interpreted 25,129 (56.3%) RDTs as positive at the time of administration, with variation across countries: the proportion positive was lower in Nigeria (39.4%) and higher in Uganda (60.4%). The number of positive results at one week was 25,022 (56.1%) and at one month was 24,864 (55.7%). Invalid results were rare with 56 (0.1%) at the time of administration, declining to 8 (0.0%) at both follow-up time points.

Bioline Malaria Pf (Abbott, IL USA) was the most commonly used RDT product (27,846, 62.4%), followed by AdvDx Malaria Pf (Advy Chemical, Mumbai, India; 6711, 15.0%) (Table 1). Benin and Nigeria used Bioline Malaria Pf and AdvDx Malaria Pf RDTs, respectively, almost exclusively whereas the majority of RDTs in Uganda were Bioline Malaria Pf but the country also used a number of other RDT products. There were 322 (0.7%) RDT products that were not recognized by the panel.

Faint lines were observed on 4246 (9.5%) RDTs at their initial administration (Table 1). The proportion varied substantially by country, with Benin reporting a much lower proportion (4.2%) and Uganda a much higher proportion (13.1%) of RDTs with faint lines. The occurrence of faint lines was also associated with the RDT product, and was more frequent among the two products with two test lines (28.9–29.9%) than among those with a single test line (7.5–12.3%). Only 6 RDTs were flagged as having blood obscuring the results window.

### Stability of results

Over the one-month evaluation period, 42,408 (95.1%) RDTs retained their original result. This included 23,901 (95.1%) of 25,129 initially positive RDTs and 18,499 (95.3%) of 19,420 initially negative RDTs. Among the 56 RDTs originally classified as invalid, 8 (14.3%) remained invalid. After one month of storage, the probability that a cassette with a positive result was originally positive (PPV) was 96.1% (95% confidence interval [CI] 95.9, 96.4) while the probability that a cassette showing a negative result was initially negative (NPV) was 93.7% (95% CI 93.4, 94.1). The equivalent probability for an invalid test result was 100% (95% CI 67.5, 100).

The proportions of RDTs that converted from positive to negative (4.9%) and negative to positive (4.7%) over one month were comparable (Table 2). No results classified initially as positive or negative changed to invalid, but 75% of the invalid tests converted to positive and 10.7% converted to negative over one month.

**Table 2:** Characteristics of rapid diagnostic tests and changes in results one week and one month after the initial interpretation (N = 44,605)

	Original to one month					Original to one week				One week to one month		
	No change in result N (%)	Positive to negative n (%)	Negative to positive n (%)	Invalid to positive n (%)	Invalid to negative n (%)	Positive to negative n (%)	Negative to positive n (%)	Invalid to positive n (%)	Invalid to negative n (%)	Positive to negative n (%)	Negative to positive n (%)	Invalid to negative n (%)
Overall	42,408 (95.1)	1228 (4.9)	921 (4.7)	42 (75.0)	6 (10.7)	1165 (4.6)	1016 (5.2)	42 (75.0)	7 (12.5)	766 (3.1)	609 (3.1)	1 (12.5)
Country												
Benin	14,923 (97.3)	291 (3.3)	119 (1.8)	1 (20.0)	0 (0.0)	294 (3.3)	115 (1.8)	2 (40.0)	0 (0.0)	108 (1.2)	115 (1.7)	0 (0.0)
Nigeria	6189 (92.1)	159 (6.0)	367 (9.0)	2 (66.7)	1 (33.3)	134 (5.1)	455 (11.2)	2 (66.7)	1 (33.3)	289 (9.7)	176 (4.7)	0 (0.0)
Uganda	21,296 (94.4)	778 (5.7)	435 (4.9)	39 (81.2)	5 (10.4)	737 (5.4)	446 (5.0)	38 (79.2)	6 (12.5)	369 (2.8)	318 (3.5)	1 (20.0)
RDT product											0 (0.0)	0 (0.0)
AdvDx Malaria Pf	6183 (92.1)	158 (6.0)	367 (9.0)	2 (66.7)	1 (33.3)	134 (5.1)	455 (11.2)	2 (66.7)	1 (33.3)	288 (9.7)	176 (4.7)	0 (0.0)
Bioline Malaria Pf	26,732 (96.0)	872 (5.4)	240 (2.1)	2 (25.0)	0 (0.0)	863 (5.3)	231 (2.0)	3 (37.5)	0 (0.0)	283 (1.8)	283 (2.3)	0 (0.0)
Bioline Malaria Pf (HRP2/pLDH)	187 (93.0)	14 (9.9)	0 (0.0)	0 (0.0)	0 (0.0)	17 (12.0)	2 (3.4)	0 (0.0)	0 (0.0)	6 (4.7)	7 (9.5)	0 (0.0)
First Response Malaria Pf	1328 (95.2)	10 (0.9)	49 (15.6)	7 (77.8)	1 (11.1)	7 (0.7)	51 (16.2)	7 (77.8)	1 (11.1)	17 (1.5)	12 (4.4)	0 (0.0)
First Response Malaria Pf Ag (pLDH/HRP2)	1756 (90.0)	21 (1.5)	139 (25.9)	31 (88.6)	4 (11.4)	21 (1.5)	139 (25.9)	30 (85.7)	5 (14.3)	42 (2.7)	43 (10.2)	0 (0.0)
ParaHIT Pf	605 (96.6)	9 (2.9)	12 (3.8)	0 (0.0)	0 (0.0)	11 (3.6)	10 (3.1)	0 (0.0)	0 (0.0)	6 (2.0)	10 (3.1)	0 (0.0)
Standard Q Pf	5320 (95.8)	133 (4.3)	100 (4.0)	0 (0.0)	0 (0.0)	105 (3.4)	116 (4.7)	0 (0.0)	0 (0.0)	117 (3.8)	73 (3.0)	0 (0.0)
Other	297 (92.2)	11 (5.4)	14 (12.1)	0 (0.0)	0 (0.0)	7 (3.4)	12 (10.3)	0 (0.0)	0 (0.0)	7 (3.3)	5 (4.5)	1 (50.0)
Faint line												
Yes	3107 (73.2)	1120 (26.7)	15 (40.5)	4 (57.1)	0 (0.0)	1057 (25.2)	15 (40.5)	4 (57.1)	0 (0.0)	296 (9.4)	233 (21.6)	1 (25.0)
No	39,301 (97.4)	108 (0.5)	906 (4.7)	38 (77.6)	6 (12.2)	108 (0.5)	1001 (5.2)	38 (77.6)	7 (14.3)	470 (2.2)	376 (2.0)	0 (0.0)

*HRP2* histidine-rich protein 2, *Pf* *Plasmodium falciparum*, *pLDH* *Plasmodium* lactate dehydrogenase, *RDT* rapid diagnostic test

**Fig. 1:**
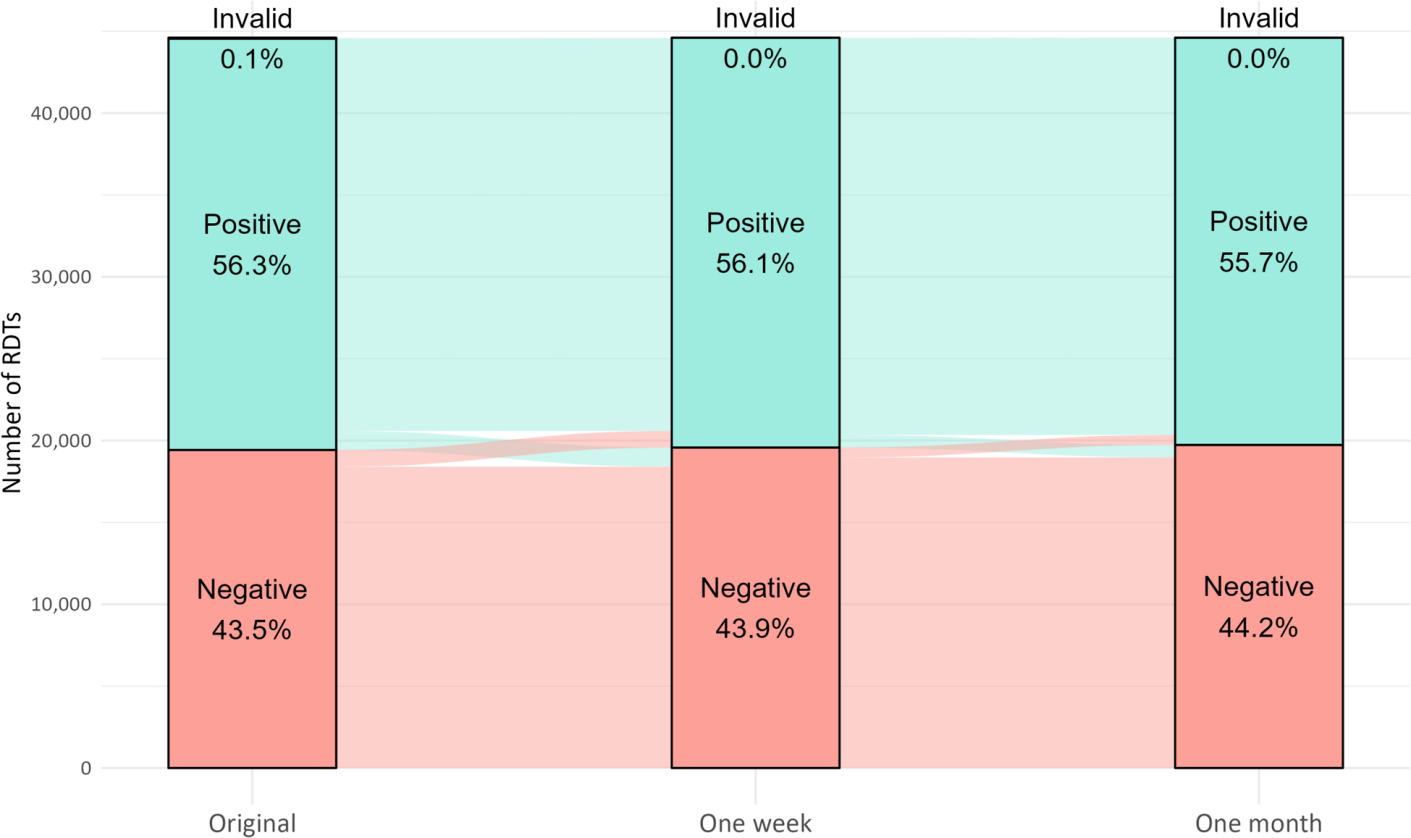
Alluvial plot showing changes in malaria rapid diagnostic test results after one week and one month of storage, 2023 (N = 44,605)

Most changes in RDT results occurred within the first week (Table 2; Fig. 1). Of the 25,129 RDTs initially interpreted as positive, 1165 (4.6%) converted to negative over the first week whereas 1016 (5.2%) of the 19,420 originally negative RDTs converted to positive (Table 2). The conversion of invalids to positive (42, 75.0%) or negative (7, 12.5%) occurred almost entirely over the first week.

**Fig. 2:**
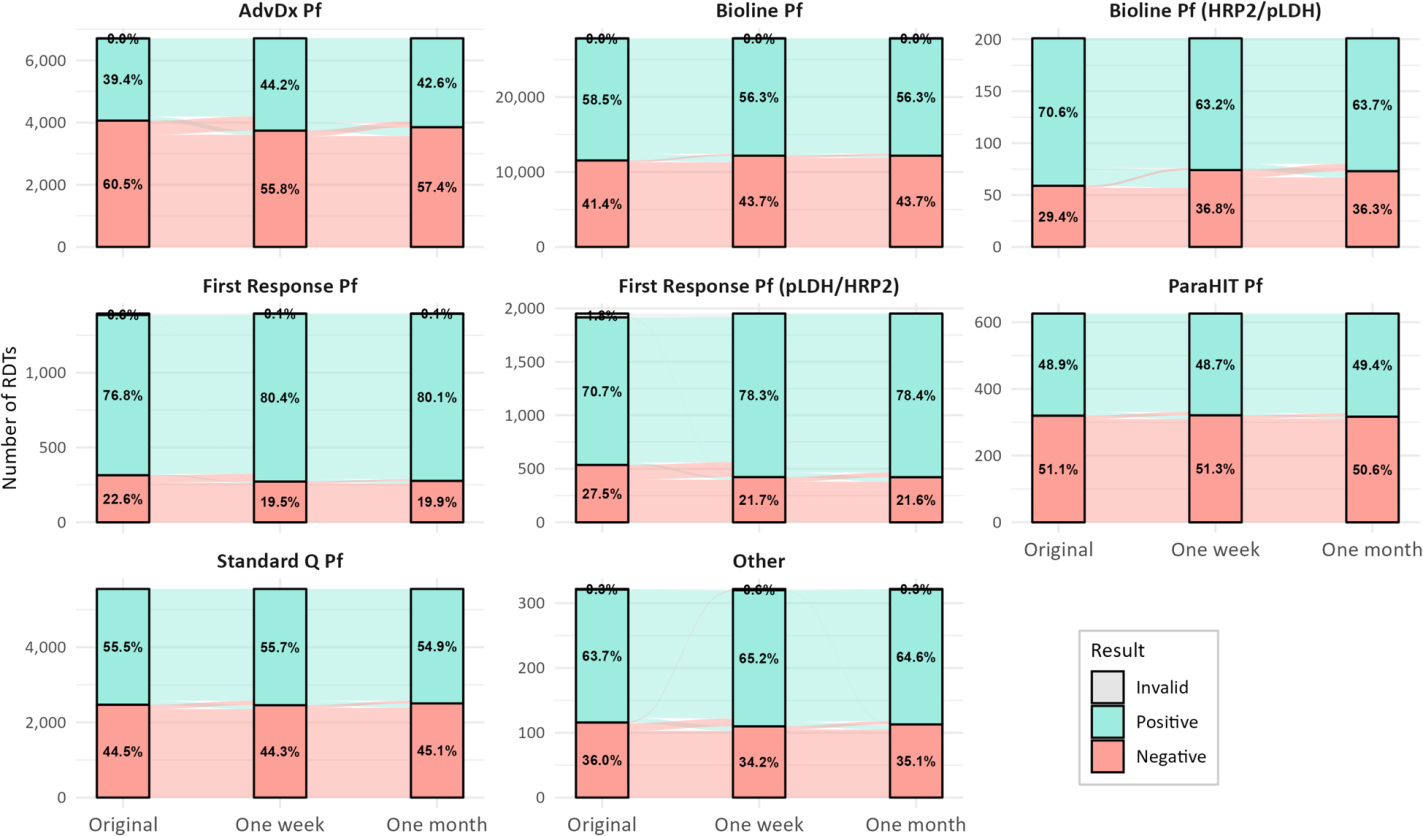
Alluvial plot showing changes in rapid diagnostic test results over one month of storage by RDT product, 2023 (N = 44,605)

The proportion of RDTs that retained their original result varied among the RDT products in the study (Fig. 2). ParaHIT Pf (Arkray Healthcare Prvt Ltd, Mumbai, India) demonstrated the most stability over one month with 96.6% of tests retaining their original result (Table 2). First Response Malaria Pf Ag (pLDH/HRP2) (Premier Medical Corporation Ltd, Gujarat, India) had the greatest proportion of tests change sign with only 90.0% retaining their original result. The Bioline Malaria Pf (HRP2/pLDH) test exhibited a higher rate of positive to negative changes (9.9%) over one month compared to the other RDTs (Table 2). In contrast, First Response Malaria Pf (0.9%) and First Response Malaria Pf Ag (pLDH/HRP2) (1.5%) tests demonstrated lower rates of conversion from positive to negative over one month. The proportion of negative RDTs converting to positive was much higher among First Response Malaria Pf Ag (pLDH/HRP2) (25.9%), First Response Malaria Pf (15.6%) and AdvDx Malaria Pf (9.0%) than other RDTs.

Faint lines were associated with large proportions of RDTs changing from positive to negative (26.8%) and negative to positive (48.1%) and only 73.2% of RDTs noted to have faint lines retained their result over one month (Table 2). Although the proportion changing from positive to negative and vice versa was much higher within one week than between one week and one month, there remained a much higher rate of change between one week and one month for the RDTs where faint lines were noted compared to those without faint lines.

**Fig. 3:**
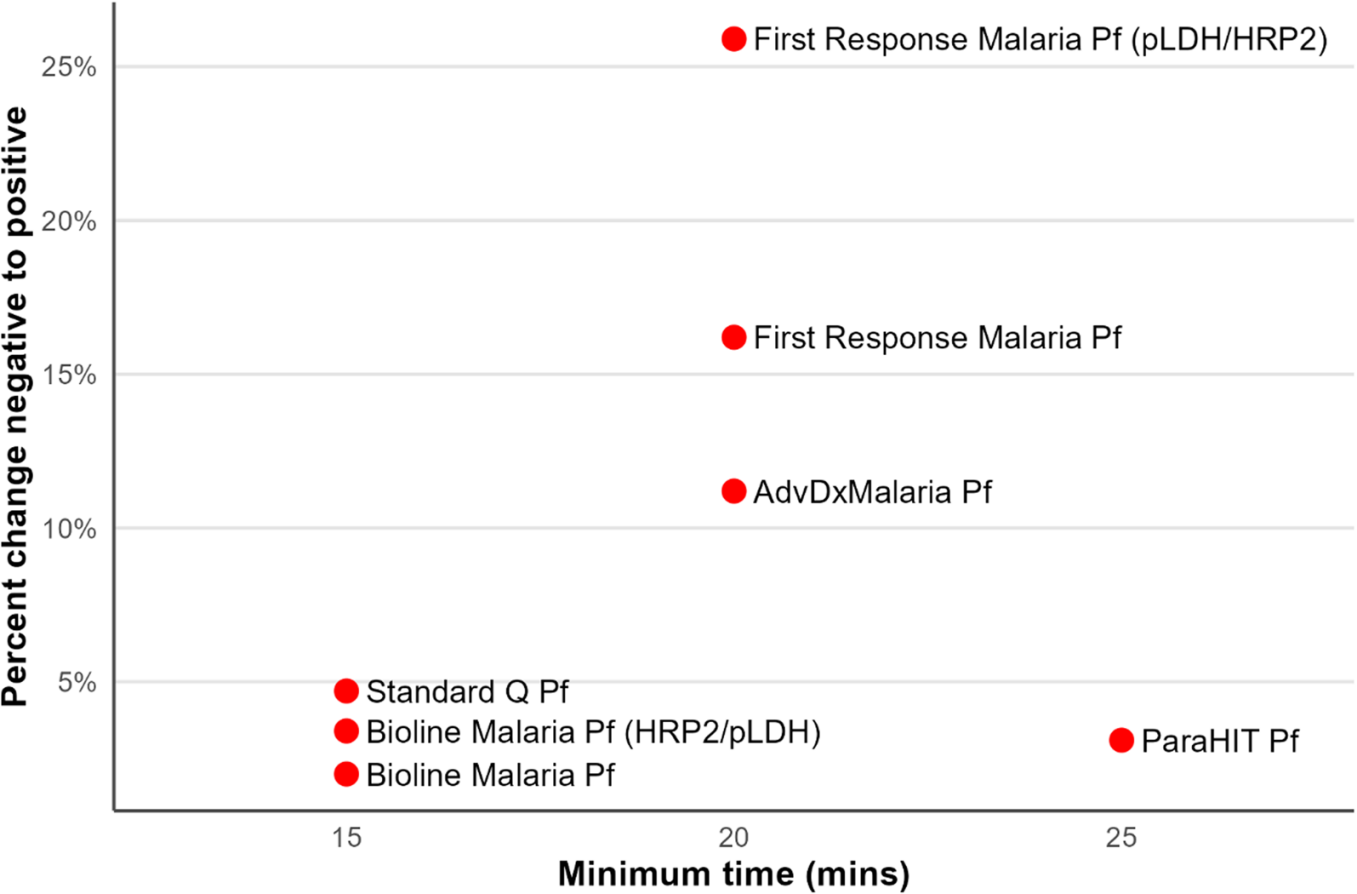
Percent change of rapid diagnostic tests from negative to positive over the first week by the minimum reading time

The study compared the proportion of initially negative RDTs that converted to positive in the first week by the minimum time recommended by the manufacturer for reading the result (Fig. 3). Three RDTs were recommended to be read between 15 and 30 min, another three between 20 and 30 min, and one test between 25 and 30 min. There was a trend toward higher proportions of tests converting to positive with longer minimum wait times, although the ParaHIT Pf test was a notable outlier with a long minimum wait time and small proportion of change.

### Stability of HRP2 and pLDH lines on two-line tests

**Fig. 4:**
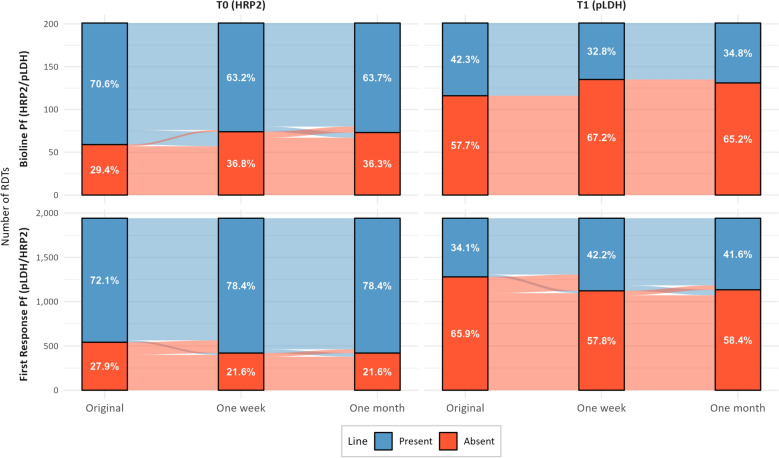
Alluvial plot showing the changes in the presence or absence of test lines in two RDT products at one week and one month

On the Bioline Pf (HRP2/pLDH) test, the T1 line detected *P. falciparum*-specific pLDH, whereas on the First Response Pf Ag (pLDH/HRP2) test, the T1 line detected pan-pLDH, which is indicative of *P. falciparum*, *P. vivax*, *Plasmodium ovale*, or *Plasmodium malariae*. For both products, changes in the T lines were more frequent within the first week than between the one-week and one-month time points (Fig. 4). Notably, the T0 (HRP2) line on the Bioline Pf test was more likely to change from present to absent during the first week, whereas the same line on the First Response test more often changed from absent to present (Table 3). This pattern was also observed for the T1 (pLDH) lines on both tests.

**Table 3:** Change in presence of test lines over one week by rapid diagnostic test product and antigen, 2023 (N = 2143)

Characteristic	No change in line presence n (%)	Present to absent n (%)	Absent to present n (%)
Bioline Malaria Pf (HRP2/pLDH) (n = 201)			
T0 (HRP2)	182 (90.5)	17 (12.0)	2 (3.4)
T1 (Pf pLDH)	182 (90.5)	19 (22.4)	0 (0)
First Response Malaria Pf Ag (pLDH/HRP2) (n = 1942)			
T0 (HRP2)	1778 (91.6)	19 (1.5)	143 (26.4)
T1 (pan-pLDH)	1732 (89.2)	26 (3.9)	184 (14.4)

*HRP2* histidine-rich protein 2, *Pf* *Plasmodium falciparum*, *pLDH* *Plasmodium* lactate dehydrogenase

## Discussion

This study found that more than 95% of stored used RDTs remained unchanged over a one-month period, with most changes occurring within the first week after test administration. Both the frequency and direction of changes in results were associated with the RDT product-type. Among products with a single test line, the AdvDx Malaria Pf demonstrated the highest overall rate of change and ParaHIT Pf showed the most stability. Both of the products with two test lines (Bioline Malaria Pf [HRP2/pLDH] and First Response Malaria Pf Ag [pLDH/HRP2]) also showed higher rates of change. AdvDx Malaria Pf and both Bioline RDT products showed higher rates of change from positive to negative compared to other products. Products showing higher rates of change from negative to positive included both First Response RDT products and AdvDx Malaria Pf.

The observed changes from negative to positive may be linked to early reading of results as there was an association between the minimum recommended read time of the RDT product type (per manufacturer instructions) and the likelihood of a change from negative to positive. It is likely that many of these RDTs were interpreted and photographed before the minimum development period had been reached. The mechanisms underlying the transition from positive to negative are less clear, but may relate to characteristics of the test membrane or the monoclonal antibodies used to detect parasite antigens.

Among RDT products with two test lines, there was no consistent association between result stability and the specific antigen target (HRP2 vs pLDH); rather, differences were more apparent at the product level. Both RDTs with two test lines observed in this study were associated with a higher proportion of tests flagged as having faint lines. Given that pLDH is generally less sensitive than HRP2 in infections where parasites express the HRP2 antigen, tests including pLDH lines may be more prone to faint bands [14]. Faint lines were associated with substantial changes in both directions, which may reflect an inherent difficulty in visualizing faint lines in photographs. Several factors may contribute to this discrepancy, including the visual acuity of the individual reading the test and lighting conditions, as photographs taken in low-light settings may benefit from camera flash, enhancing line visibility. However, the most critical factor appears to be the degree of attention devoted to interpretation: faint lines are more likely to be overlooked during a cursory glance but are often detected when the reader takes the time to carefully examine the result.

RDTs are primarily designed to provide point-of-care parasitological confirmation of malaria and guide treatment decisions. Their widespread scale-up over the past decade has additionally strengthened malaria surveillance by expanding coverage of laboratory testing and standardizing diagnostic confirmation. Increasingly, RDTs are also used in research, including for parasite DNA extraction and genomic analysis [15, 16]. However, to the authors’ knowledge, this represents the first analysis of RDT result stability to assess their potential as a source record for cross-verifying data in health facility registers, laboratory registers, and national health information systems. National malaria programmes have already begun to use stored RDTs to cross-check health facility records to rationalize use of antimalarial medicines and strengthen data quality [11, 17]. This study demonstrates that there is a 96.1% probability that a cassette indicating a positive result after being stored for one month was initially positive and a 93.7% probability that a cassette indicating negative at one month was initially negative, providing reasonable confidence that RDT cassettes can be used as a reference standard to compare with health facility records of RDT results. The findings from this study, therefore, generally support the use of malaria RDTs stored for up to one month under typical ambient conditions in health facilities for data validation purposes, in line with the approach implemented in Benin. However, the specific RDT product used will affect the validity of this approach and countries are recommended to review these results and take the RDT product into account when deciding whether to validate health facility data with stored RDT cassettes. Results from this study can also be used to determine the sample size that would be needed to evaluate other RDT products not observed in this study. Finally, further research assessing the stability of RDT results beyond one month (such as over a three-month period), could help determine the feasibility of longer storage durations, potentially providing a more practical and cost-effective frequency for routine data verification.

A key strength of this study was the large number of RDTs observed and the inclusion of multiple RDT products across three countries. The use of barcodes to track individual RDTs enabled systematic follow-up and image capture over a one-month period. However, a notable limitation was the absence of follow-up images for over 9,000 RDTs. While many of these were likely missing due to the time constraints of the study, there was evidence that RDTs initially interpreted as negative were more likely to be missing follow-up images. This may reflect a tendency among health facility staff to prioritize the retention of positive tests, although we do not believe this would systematically bias the assessment of result stability over time as the rate of change was similar. Additionally, faint test lines continue to pose a challenge for visual interpretation of RDTs. It is likely that a proportion of RDTs with faint lines were misclassified, which could have affected the results for certain RDT products, particularly those with pLDH lines. The used RDTs were stored at ambient conditions in health facilities throughout the study. Although storage temperature was not monitored, it is recognized that prolonged high temperatures can affect the performance of RDTs [18]. Further studies examining the impact of temperature on the stability of used RDT results would help guide expectations under different environmental conditions.

## Conclusions

This study demonstrated that more than 95% of stored RDT cassettes retained their original result. Close to half of the changes may be due to reading RDTs before their period of development has concluded. Health officials should emphasize the need to follow manufacturer’s guidelines and ensure that tests are only interpreted as negative after the minimum development time has elapsed. Despite this finding, RDT cassettes stored for one month provide reliable information on the original RDT result and could be used as part of a strategy to evaluate surveillance data quality and the rational use of antimalarials in health facilities in Africa.

## Supplementary Information

Additional file 1.

## Data Availability

The datasets used and/or analysed during the current evaluation can be provided by the corresponding author on reasonable request.

## Abbreviations

CI: Confidence interval
HRP2: Histidine-rich protein 2
LTFU: Lost to follow-up
Pf: *Plasmodium falciparum*
pLDH: Plasmodium lactate dehydrogenase
PMI: President’s Malaria Initiative
RDT: Rapid diagnostic test
WHO: World Health Organization
