# Supplementary material for: Are malaria rapid diagnostic test results stable over time to support verification of surveillance data?
Source: Malar J. 2025 Oct 22;24:356. doi: 10.1186/s12936-025-05595-0 (PMC12542119; doi:10.1186/s12936-025-05595-0)
Supplement: Supplementary file 1 — Additional file 1. [file 12936_2025_5595_MOESM1_ESM.docx]

Supplementary table 1. Description of RDT products observed in the MaCRA study.

| **Test name (manufacturer)**  **Result interpretation time period** | **Referred to as:**  **Result interpretation window** | **RDT cassette format** |
| --- | --- | --- |
| RDT cassettes with a single test line to detect *Plasmodium falciparum*-specific histidine-rich protein 2 (HRP2) | | |
| AdvDx Malaria Pf Rapid Malaria Ag Detection Test (Advy Chemical, Mumbai, India)  20 – 30 minutes | AdvDx Malaria Pf | 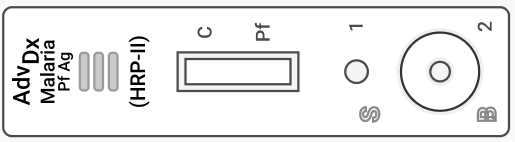 |
| Bioline MALARIA Ag P.f  (Abbott, IL USA)  *Note: The same cassette previously was manufactured as SD Bioline. Both versions of the RDT appeared in this study.*  15 – 30 minutes | Bioline Malaria Pf | 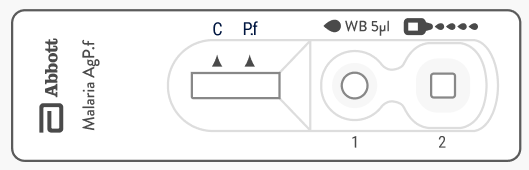  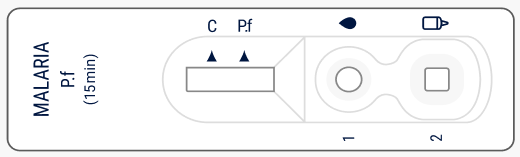 |
| First Response Malaria Antigen *P. falciparum* (HRP2) Card Test (Premier Medical Corporation Ltd, Gujarat, India)  20 – 30 minutes | First Response Malaria Pf | 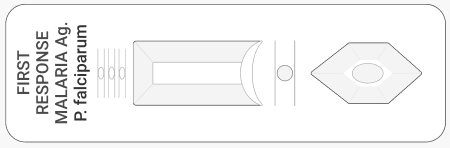 |
| ParaHIT f Ver. 1.0 Rapid Test for *P. falciparum* Malaria Device  (Arkray Healthcare Prvt Ltd, Mumbai, India)  25 – 30 minutes | ParaHIT Malaria Pf | 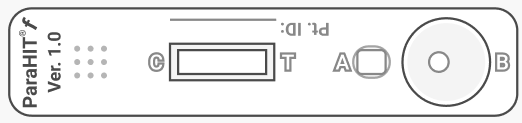 |
| STANDARD Q Malaria P.f Ag Test (SD Biosensor, Gyeonggi-do, Republic of Korea)  15 – 30 minutes | Standard Q Malaria Pf | 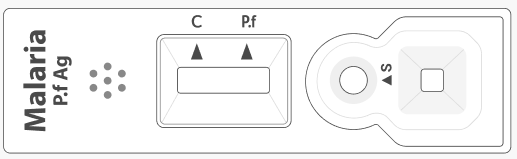 |
| RDT cassettes with two test lines to detect *Plasmodium falciparum* HRP2 and *Plasmodium falciparum*-specific lactate dehydrogenase (pLDH) | | |
| Bioline Malaria Ag P.f (HRP2/pLDH) (Abbott, IL USA)  15 – 30 minutes | Bioline Malaria Pf (HRP2/pLDH) | 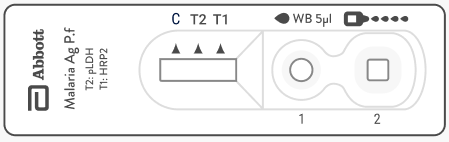 |
| RDT cassettes with two test lines to detect *Plasmodium falciparum* HRP2 and pan-pLDH | | |
| First Response Malaria Ag. pLDH/HRP2 Combo Card Test (Premier Medical Corporation Ltd, Gujarat, India)  *Note: pLDH line does not distinguish between* P. falciparum *and non-*falciparum *species*  20 – 30 minutes | First Response Malaria Pf Ag (pLDH/HRP2) | 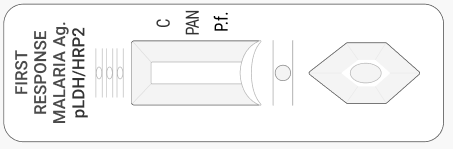 |
